# Vascular endothelial growth factor immunoexpression in oral paracoccidioidomycosis

**DOI:** 10.4317/medoral.26886

**Published:** 2025-01-26

**Authors:** Elisângela de Souza Santos Dias, Larissa Couto de Freitas, Marta Miyazawa, Denismar Alves Nogueira, Carine Ervolino de Oliveira, Alessandro Antônio Costa Pereira, João Adolfo Costa Hanemann

**Affiliations:** 1 School of Dentistry. Federal University of Alfenas. Alfenas, MG, Brazil; 2 Institute of Exact Sciences. Federal University of Alfenas. Alfenas, MG, Brazil; 3 Departament of Pathology and Parasitology. Institute of Biomedical Sciences. Federal University of Alfenas. Alfenas, MG, Brazil

## Abstract

**Background:**

Paracoccidioidomycosis (PCM) is a systemic mycosis endemic and limited to Latin America. Brazil is responsible for more than 80% of diagnosed cases in the world. Since PCM is not a notifiable disease, there are still no accurate data on its incidence in Brazil. Vascular endothelial growth factor (VEGF) is considered the main vascular endothelial growth factor expressed in the process of angiogenesis, both under physiological and pathological conditions. To date, there are not studies in the literature that evaluated the expression of VEGF in oral PCM lesions. Therefore, the objective of this study was to evaluate the prevalence of oral lesions of PCM diagnosed from 1998 to 2020; to analyze the immunoexpression of VEGF in oral lesions of PCM; and to compare the VEGF immunostaining with the clinical and microscopic aspects of these lesions.

**Material and Methods:**

Clinical data of 98 cases of patients with oral PCM were evaluated. A total of 41 selected cases were quantitatively and qualitatively analysed by immunohistochemistry for VEGF.

**Results:**

Our results showed that oral PCM preferentially affects white males, with mean age of 50.2 years, and the gingiva and the alveolar ridge. It was not possible to correlate VEGF immunoexpression with clinical and microscopic variables.

**Conclusions:**

Oral PCM is a relatively uncommon pathological condition and that, in our sample, the immunoexpression of VEGF was mild and observed in a reduced number of cases.

** Key words:**Paracoccidioidomycosis, Paracoccidioides brasiliensis, VEGF, angiogenesis, immunohistochemistry, histopathology, biopsy, oral mucosa.

## Introduction

Paracoccidioidomycosis (PCM) is a systemic granulomatous fungal infection caused by inhalation of the thermodimorphic fungus Paracoccidioides (1-2). The infection is autochthonous and restricted to the American continent, and Brazil is the largest holder of PCM cases, with high incidence in the South and Southeast regions (3). PCM represents the main cause of disability and death among young adult rural workers, aged between 30 and 50 years, during the most productive period of life. Both genders can be infected, however there is higher predilection for males, with a male-female ratio of 11:1 (4).

The oral lesions are characterized by the presence of an erythematous granular hyperplasia, with purplish hemorrhagic spots and a rough surface appearance called “moriform stomatitis” (5,6). On the lips, it leads to the appearance of macrocheilia, represented by the pronounced increase in thickness and consistency of the lips. In general, oral lesions are multiple and localized on the lips, gingiva, buccal mucosa, palate, tongue and floor of the mouth. Although uncommon, PCM can cause perforation of the hard palate (5). Microscopically, oral PCM lesions present pseudoepitheliomatous hyperplasia, intraepithelial microabscesses, granulomatous reaction composed of inflammatory multinucleated giant cells and predominantly mononuclear intense inflammatory infiltrate (lymphocytes and plasma cells), and mainly with spherical structures with a birefringent wall, corresponding to the morphology of the fungus Paracoccidioides (6-8). The treatment of this disease is based on the administration of antifungals for approximately 12 to 24 months, depending on the clinical manifestation of the disease (9).

Angiogenesis is an essential process for the formation of new blood vessels from preexisting vessels. It is a complex phenomenon involving numerous molecules and growth factors that will stimulate and/or inhibit neovascularization (10,11). The formation of new blood vessels is essential for the transport of nutrients to tissues and organs. However, the imbalance of this process may lead to the development of several pathological processes (11-13). This angiogenic process is controlled by several endothelial growth factors, among which the Vascular Endothelial Growth Factor (VEGF) stands out (10).

In periodontal disease, studies have revealed nuclear and cytoplasmic immunoreactivity in inflammatory and endothelial cells, suggesting that VEGF is an important factor in the initiation and progression of this oral disease due to the association of increased inflammation and the ability to promote vascular expansion and angiogenesis (14). Ferreira *et al* (15) evaluated the effect of low-level laser irradiation (Helium-Neon) on extracellular matrix deposition, cytokine expression and angiogenesis in experimental paracoccidioidomycotic lesions. The results showed that the irradiated lesions expressed VEGF in the blood vessels, whereas in the untreated lesions, VEGF expression was observed only in a small area in the center of the lesions. This suggests that, although hypoxia is considered an important triggering factor for the transcription of angiogenic factors, severe, prolonged, tissue-spread hypoxia in non-irradiated lesions was able to impair the healing process of these wounds. On the other hand, the laser stimulated aerobic cell metabolism and accelerated tissue repair.

To date, no studies were found in the literature evaluating the expression of VEGF in oral PCM lesions. Therefore, the objective of this study was to evaluate the prevalence of oral lesions of PCM diagnosed from 1998 to 2020; to analyze the immunoexpression of VEGF in oral lesions of PCM; and to compare the VEGF immunostaining with the clinical and microscopic aspects of these lesions.

## Material and Methods

- Patients

This research project was approved by the Research Ethics Committee involving human beings of Federal University of Alfenas (CAAE 40286520.1.0000.5142). It was carried out the survey of all oral lesions of PCM diagnosed and filed at the Oral Pathology Service of the Federal University of Alfenas (Alfenas, MG, Brazil) from 1998 to 2020. The demographic data of the patients were collected, such as gender, age, ethnicity, profession, harmful habits related to the oral cavity (smoking and alcohol consumption), the location of the lesion, the clinical aspect and the suggested clinical diagnosis by the professional.

- Qualitative microscopic analysis

For the microscopic analysis, 5 µm thick sections were stained with Hematoxylin and Eosin (HE). Morphological analysis of the lesions was performed individually by two examiners using a binocular optical microscope (AxioLab-Carl Zeiss, Göttingen, Germany) containing a 40X/0.65 N-Achroplan objective lens, resulting in a final magnification of 400X.

For the microscopic analysis, the following histopathological criteria were used: 1) Presence of pseudoepitheliomatous hyperplasia; 2) Presence of areas of intraepithelial microabscesses; 3) Presence of granulomatous reaction consisting of inflammatory multinucleated giant cells (IGMCs) and inflammatory infiltrate containing mononuclear and polymorphonuclear cells; and 4) Presence of spherical structures with a birefringent cell wall corresponding to the fungus (6).

In addition to these diagnostic microscopic changes, it was also analyzed the intensity and location of the inflammatory infiltrate, the type of inflammatory MGCs (Langhans or foreign body) and the location of the fungi: dispersed in the connective tissue, inside the MGCs or in the areas of microabscesses.

- Immunohistochemistry

Three micrometers (µm) thick sections were placed on silanized slides for the immunohistochemical reaction. The microscopic sections were dewaxed in xylene, performing two exchanges of five minutes each. Then, they were hydrated in alcohols in decreasing concentrations: absolute; 90%; 70%; 50%; until running water and then washed in running water for five minutes.

Next, 420 mL of distilled water were placed in the recovery chamber of the Pascal Pan at 118°C for two minutes. It was allowed the temperature to reach 90°C and after 10 seconds the remaining pressure was removed. Then, the lid of the pan was opened and the vat with the blades was placed at room temperature for 20 minutes. The sections were then washed and kept in distilled water until the next step.

Endogenous peroxidase was inactivated with 3% hydrogen peroxide in PBS for 10 minutes, after which the sections were washed with distilled water. VEGF primary antibody (M7273, clone VG1; Dako, Carpinteria, CA, USA; dilution 1:150) was applied and the sections placed in a humidified chamber in a refrigerator at 4°C overnight. After incubation with the primary antibody, the slides were washed with PBS. After washing, the binding polymer (EnViosion Flex HRP. Dako, Carpinteria, CA, USA) was applied and left for 40 minutes at room temperature. Then, the slides were washed with PBS, the excess was aspirated and diaminobenzidine (Dako, Glostrup, Denmark) was applied as chromogen for 10 minutes at room temperature. Counterstaining was performed with Harris Hematoxylin for 2 minutes. Positive and negative controls, by omitting the primary antibody, were used in all reactions.

- VEGF immunostaining score

Semi-quantitative immunohistochemical analysis was performed according to Klein *et al* (16). Initially, it was analyzed the percentage of labeled cells: Score 0 = absence of labeling; Score 1 = up to 30% of labeled cells; Score 2 = between 30 and 60% of labeled cells; Score 3 = more than 60% of labeled cells. Then, the intensity of labeling was evaluated, where 0 = absence of staining; 1 = weak staining; 2 = moderate staining; 3 = strong staining. To determine the final staining scores, the score of the percentage of labeled cells was multiplied by the intensity of the staining, which values ranged from 0 to 9. This analysis was performed by two independent examiners (17).

- Statistical analysis

To compare the categorical variables in contingency Tables, the chi-square frequency test was adopted and when an expected frequency was lower than 5, Fisher's exact test was applied. A significance level of 5% (*p* ≤ 0.05) was considered for the statistical tests.

## Results

**Figure 1:**
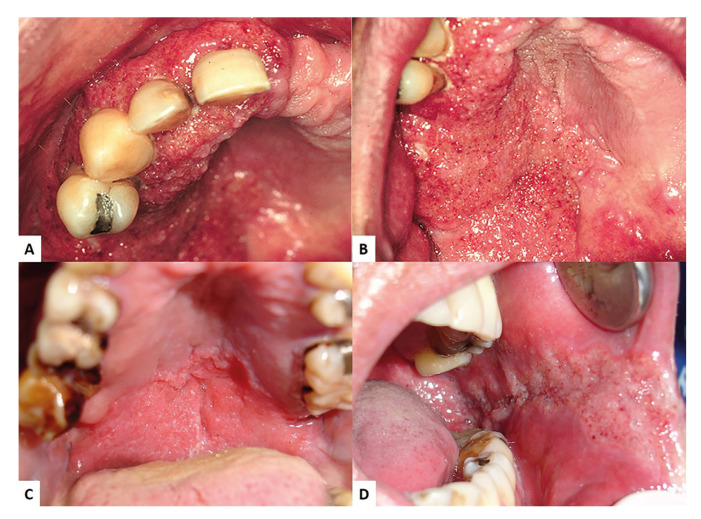
Moriform stomatitis in gingiva/alveolar ridge (A), palate (B-C), and buccal mucosa (D).

From a total of 10.667 anatomopathological reports issued between January 1998 and December 2020, there were 98 (0.92%) cases of oral PCM diagnosis. PCM affected more frequently the male gender (76.5%). The age of patients ranged from 21 to 80 years, with a mean age of 50.2 years. Most of cases were diagnosed in the fourth and fifth decades of life. Regarding the location, 59 (28.5%) patients developed lesions of PCM in the gingiva and/or alveolar ridge (Fig. 1). The palate (Fig. 1) was affected in 28 cases (13.5%) and the buccal mucosa (Fig. 1), in 24 cases (11.6%). The protocol of treatment adopted in our service for PCM is itraconazole 200 mg/day during 6 to 12 months. All patients showed complete remission of oral lesions after completion of the treatment.

**Figure 2:**
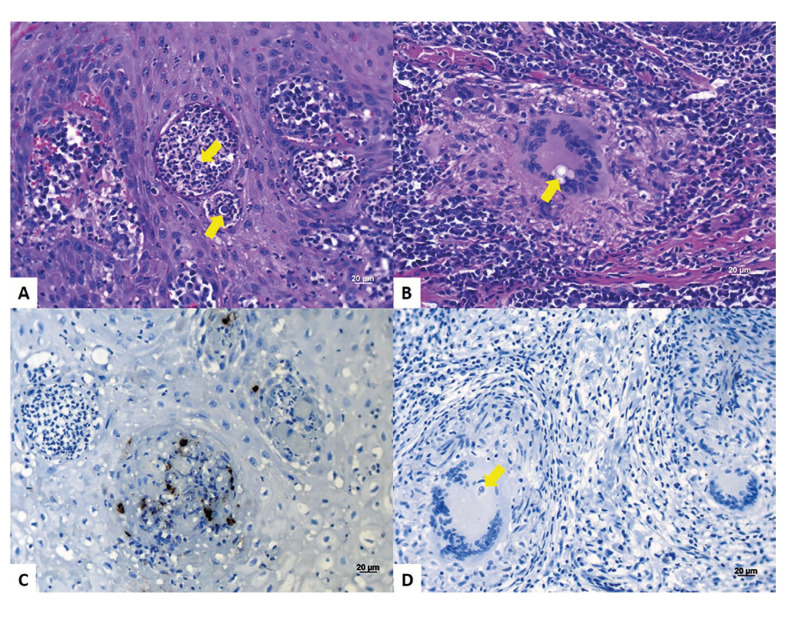
<italic>Paracoccidioides brasiliensis</italic> fungi (arrows) were present in the areas of microabscesses (A), and inside the IGMCs (B). Macrophage-compatible VEGF-immunostained cells (C). Absence of VEGF immunostaining in fungi (arrow) located inside Langhans-type IGMCs (D).

Microscopic analysis was performed in 83 cases (84.6%). Granulomas, when present, followed the classification proposed by Abreu e Silva *et al* (7) and were divided into immunogenic (dense) and non-immunogenic (loose). Among analyzed cases, it was observed pseudoepitheliomatous hyperplasia in 78 cases (94%), microabscesses in 74 cases (89%), mononuclear inflammatory infiltrate in all 83 cases (100%), IGMCs in 78 cases (94%), and immunogenic granulomas in 32 cases (39%). Regarding the intensity and location of the inflammatory infiltrate, in the vast majority of cases showed intense and diffusely located through the connective tissue. In general, fungi were present dispersed throughout the connective tissue, in the areas of microabscesses (Fig. 2), and inside the IGMCs (Fig. 2).

A total of 41 cases were selected for immunohistochemical analysis. Of these, only 10 cases showed immunopositivity for VEGF, and in seven cases (70%) the staining was mild, in two cases (20%) intense, and in only one case (10%) it was moderate. As for the amount of labeled cells, all 10 cases had less than 30% of the immunostained cells. When the scores obtained by the intensity of immunostaining were multiplied by the scores represented by the percentage of immunostained cells, seven cases (70%) had a final score of “1”, two cases (20%) had a score of “3” and only one case (10%) had score “2”. Clinical and VEGF microscopical analysis data are shown on Table 1 and the unknown data was left blank.

There was a predominance of immunostaining in macrophages (Fig. 2), in the vascular wall and more intensely in areas of serofibrinous pseudomembrane. No staining was observed in the fungi *Paracoccidioides brasiliensis* (Fig. 2). In addition, immunopositivity was also observed in the intravascular plasma.

When correlations were performed between the clinical variables (age, gender, ethnicity, location and clinical aspects of the lesions) and the microscopic variables, no statistical significance was observed. It was not possible to correlate VEGF immunoexpression with clinical and microscopic variables, considering that only 10 cases were immunopositive for VEGF, making statistical analysis unfeasible.

## Discussion

Paracoccidioidomycosis (PCM) is a systemic mycosis related to agricultural activities with an underestimated incidence and prevalence due to the lack of notification in several Brazilian Federation Units. The insidious evolution of the clinical condition can result in serious sequelae if diagnosis and treatment are not early and properly instituted (18). This condition is endemic and limited to Latin America. Brazil is responsible for more than 80% of diagnosed cases in the world and the most prevalent Brazilian regions are Southeast, Midwest and South (19). Since PCM is not a notifiable disease, there are still no accurate data on its incidence in Brazil (20). Therefore, epidemiological studies are of great importance to understand the real prevalence of this disease.

In this study we analyzed the prevalence of PCM oral lesions diagnosed in the period from 1998 to 2020. In all cases, patients presented PCM mucosal lesions where oral manifestations were the reason for the first appointment, as defined by Verli *et al* (3). Our results revealed that, from a total of 10.667 anatomopathological reports issued in this period, 98 (0.92%) cases of oral PCM were diagnosed, similarly (0.65%) to that observed by Brazão-Silva *et al* (21). In a recent multicenter study conducted by Arruda *et al* (22), the 320 cases diagnosed as PCM represented only 0.3% of the total oral and maxillofacial lesions. This is probably due to the fact that the diagnostic centers that provided cases for the study are located in different regions of the country.

Regarding demographic aspects, in our series there was a predominance of male patients, aged between 21 and 80 years, with an average age of 50.2 years. In addition, it was observed that the greatest number of cases were diagnosed in the fourth and fifth decades of life. These results are in accordance to those reported by Arruda *et al* (22), who found a predominance of male patients (93.4%) with a mean age of 51.3 years. The age group most affected by PCM was between the fifth and sixth decades of life. Brazão-Silva *et al* (21) also found a predominance of patients in the fourth to sixth decades of life, with a mean age of 45.2 years, as well as a high male predilection (male/female ratio of 15.5:1). It is noteworthy that these two studies included patients younger than 20 years of age, probably diagnosed with juvenile PCM, which differs from our series, which included only patients with the chronic form of PCM. In addition, Bicalho *et al* (23) showed a greater predominance of male involvement (ratio of 30:1) and a slightly lower mean age (40 years).

Oral lesions are found in a percentage generally above 80% of patients with PCM and, in most cases, these lesions represent the first sign and/or symptom that motivates the patient to look for a health professional (21). Oral lesions are typically characterized by moriform stomatitis, periodontal involvement and, eventually, the presence of vegetating and ulcerative-vegetative lesions (6). Another aspect frequently observed in patients with oral PCM is macrocheilia (24). The involvement of the oral mucosa by PCM is usually multifocal, that is, different regions can be affected simultaneously (3). The most prevalent regions are the gingiva/alveolar ridge, the labial and buccal mucosa, tongue and floor of the mouth (21-23,25). In our series, we also observed a marked predominance of involvement of the gingiva/alveolar ridge, followed by palate, oropharynx, and buccal mucosa (Table 1). As for the clinical presentations of oral PCM reported by the professionals contained in the biopsy records, moriform stomatitis was the main reported clinical presentation.

Oral lesions in all forms of PCM are microscopically characterized by the formation of granulomas with inflammatory MGCs and epitheliod macrophages, in addition to lymphocytes, plasmocytes and neutrophils (7,26). The lining epithelial tissue has an ulcerated surface as well as pseudoepitheliomatous hyperplasia. Fungi are usually found inside IGMCs, dispersed through connective tissue or in areas of microabscesses. Granulomas can be dense or loose (7,27-29). In our study, the microscopic aspects observed are in accordance with what has been reported over the years in the literature and, in particular, by our previous studies (4,6,8,28,29).

VEGF is considered the main vascular endothelial growth factor expressed in the process of angiogenesis, both under physiological and pathological conditions (10). Angiogenesis is present in several processes of the organism such as tissue growth, inflammation, healing and immune response; and, it is also involved in the etiopathogenesis of several diseases (12,13), including oral lesions such as periodontal disease (14). In our study, a predominance of immunostaining by VEGF was observed in inflammatory cells compatible with macrophages and also in the intravascular plasma.

To date, we have not found studies in the literature that evaluated the expression of VEGF in oral PCM lesions. Ferreira *et al* (15) demonstrated a strong expression of VEGF in the vascular walls of tissues irradiated with low-level laser therapy and concluded that photobiomodulation was efficient to minimize the local effects caused by *Paracoccidioides brasiliensis*, accelerating the wound healing process and, consequently, reducing the patient's discomfort and pain. Moreover, our group demonstrated for the first time in the literature the effectiveness of photodynamic therapy as an adjuvant in the treatment of PCM oral lesions by significantly reducing morbidity and improving the quality of life of patients (30).

In our study, it was not possible to correlate VEGF immunoexpression in oral PCM lesions with clinical and microscopic aspects. Therefore, further studies should be performed using other angiogenesis markers, such as CD31 and CD34 to better understand this process and the different clinical manifestations of the disease. The understanding of the action of VEGF in the tissues of the oral mucosa affected by PCM may lead to the development of new therapeutics by accelerating the repair process of oral lesions, reducing morbidity and improving the quality of life of patients.

In conclusion, the oral PCM is a relatively uncommon pathological condition. It preferentially affects men, aged between 40 and 60 years old, Caucasian, and frequently located in the gingiva and alveolar ridge, and predominantly presented as moriform stomatitis. In our sample, VEGF immunoexpression was mild and observed in a reduced number of cases.

## Figures and Tables

**Table 1:** Clinical data of 41 patients and its VEGF immunoexpression analysis. The unknown data was left blank.

#	Age	Gender	Localization					Clinical Aspect	VEGF immunoexpression		
			Lip	Tongue	Gingiva/ alveolar ridge	Palate, oropharynx	Buccal mucosa		I	Q	IxQ
1	42	M	X	-	-	-	X	moriform stomatitis	0	0	0
2	42	M	-	-	X	-	-	moriform stomatitis	1	1	1
3	31	M	-	-	-	X	-	-	0	0	0
4	48	M	-	X	-	X	-	moriform stomatitis	0	0	0
5	46	M	X	-	-	X	X	moriform stomatitis	0	0	0
6	56	M	-	-	-	-	-	moriform stomatitis	0	0	0
7	46	M	X	-	-	-	X	moriform stomatitis	0	0	0
8	45	M	-	-	-	X	X	moriform stomatitis	3	1	3
9	57	M	-	-	X	-	-	moriform stomatitis	0	0	0
10	55	M	-	X	-	-	-	moriform stomatitis	0	0	0
11	59	M	-	-	X	-	-	moriform stomatitis	0	0	0
12	54	M	-	-	-	X	-	moriform stomatitis	0	0	0
13	49	M	X	-	-	-	X	moriform stomatitis	0	0	0
14	44	M	X	X	-	-	X	moriform stomatitis	0	0	0
15	65	M	X	-	-	-	-	moriform stomatitis	0	0	0
16	55	M	-	-	X	-	-	moriform stomatitis	0	0	0
17	63	M	X	X	-	-	X	moriform stomatitis	0	0	0
18	58	M	X	-	-	-	-	-	0	0	0
19	50	M	-	-	-	X	-	moriform stomatitis	0	0	0
20	37	M	X	-	-	X	X	moriform stomatitis	0	0	0
21	40	F	-	-	X	-	-	moriform stomatitis	2	1	2
22	47	M	-	-	-	-	X	moriform stomatitis	1	1	1
23	42	F	-	X	-	-	-	moriform stomatitis	0	0	0
24	40	M	-	X	-	-	X	moriform stomatitis	0	0	0
25	-	F	-	-	X	-	-	moriform stomatitis	0	0	0
26	47	M	-	-	-	-	X	moriform stomatitis	0	0	0
27	39	F	-	-	-	X	-	moriform stomatitis	0	0	0
28	48	F	X	-	-	X	-	moriform stomatitis	0	0	0
29	48	M	-	-	-	X	X	moriform stomatitis	0	0	0
30	49	M	-	-	-	-	X	vegetating ulcer	1	1	1
31	44	M	X	-	-	-	-	moriform stomatitis	0	0	0
32	56	M	-	-	-	X	-	moriform stomatitis	1	1	1
33	54	M	-	X	-	-	-	moriform stomatitis	0	0	0
34	43	M	-	-	X	-	-	periodontitis	3	1	3
35	51	F	X	-	-	-	-	moriform stomatitis	0	0	0
36	78	M	-	-	X	-	-	moriform stomatitis	1	1	1
37	67	M	-	-	X	-	-	moriform stomatitis	0	0	0
38	70	F	X	-	-	X	X	moriform stomatitis	1	1	1
39	69	M	-	-	-	X	-	moriform stomatitis	0	0	0
40	47	F	-	-	-	X	-	moriform stomatitis	1	1	1
41	49	M	-	-	-	X	-	moriform stomatitis	0	0	0

M: male; F: female; I: intensity; Q: quantification.
